# Pleural drainage vs video-assisted thoracoscopic debridement in children affected by pleural empyema

**DOI:** 10.1007/s00383-023-05566-z

**Published:** 2023-11-06

**Authors:** Alberto Ratta, Francesca Nascimben, Rossella Angotti, Camilla Todesco, Veronica Carlini, Giulia Fusi, Lorenzo De Biagi, Simona Straziuso, Francesco Italiano, Vincenzo Domenichelli, Mario Messina, Francesco Molinaro

**Affiliations:** 1 Pediatric Surgery Unit, Infermi Hospital, AUSL Romagna, Rimini, Italy; 2 Division of Paediatric Surgery, Department of Medical, Surgical and Neurological Sciences, Policlinico Le Scotte, University of Siena, Viale Bracci 14, 53100 Siena, Italy

**Keywords:** Pleuric empyema, Thoracic drainage, VATS, Ultrasound, Children

## Abstract

**Background:**

Both thoracic drainage and video-assisted thoracic surgery (VATS) are available treatment for pleural empyema in pediatric patients.

**Materials and methods:**

This retrospective multicenter study includes pediatric patients affected by pleural empyema treated from 2004 to 2021 at two Italian centers. Patients were divided in G1 (traditional approach) and G2 (VATS). Demographic and recovery data, laboratory tests, imaging, surgical findings, post-operative management and follow-up were analyzed.

**Results:**

70 patients with a mean age of 4.8 years were included; 12 (17.1%) in G1 and 58 (82.9%) in G2. Median surgical time was 45 min in G1, 90 in G2 (*p* < 0.05). Mean duration of thoracic drainage was 7.3 days in G1, 6.2 in G2 (*p* > 0.05). Patients became afebrile after a mean of 6.4 days G1, 3.9 in G2 (*p* < 0.05). Mean duration of antibiotic therapy was 27.8 days in G1, 25 in G2 (*p* < 0.05). Mean duration of postoperative hospital stay was 16 days in G1, 12.1 in G2 (*p* < 0.05). There were 4 cases (33.3%) of postoperative complications in G1, 17 (29.3%) in G2 (*p* > 0.05). 2 (16.7%) patients of G1 needed a redosurgery with VATS, 1 (1.7%) in G2.

**Conclusions:**

VATS is an effective and safe procedure in treatment of Pleural Empyema in children: it is associated to reduction of chest tube drainage, duration of fever, hospital stay, time of antibiotic therapy and recurrence rate.

**Supplementary Information:**

The online version contains supplementary material available at 10.1007/s00383-023-05566-z.

## Introduction

Pleural empyema is a common disease in children, especially in case of severe comorbidities [1]. The pathogenesis is not completely clear, but it seems to be due to a primary pneumonia with a consequent bacterial migration towards the pleural space [1]. About 0.6–2% of pediatric cases of pneumonia evolves into empyema, with an incidence of 3.3 on 100.000 children [2]. Empyema can be divided in three stages: Stage 1 or Exudative, Stage 2 or Fibrino-purulent stage and Stage 3 or Organizing stage [3]. Nowadays, there are several therapeutic strategies such as thoracic drainage and fibrinolysis, thoracotomy and Video Assisted Thoracic Surgery (VATS) to treat pleural empyema. [4]. Even if thoracoscopic debridement brought an innovation in the treatment of pleural empyema in children, there is still no consensus about which is the gold standard in pediatric population [4].

## Aim of the study

The aim of the study is to make a comparison between conservative approaches such as thoracic drainage and Video-Assisted Thoracic Surgery (VATS) in the treatment of pleural empyema in pediatric patients through the analysis of a multicenter experience.

## Materials and methods

This retrospective multicenter cohort study includes all pediatric patients younger than 16 years old affected by pleural empyema treated at the Pediatric Surgery Department of University Hospital of Siena and the Pediatric Surgery Unit of AUSL Romagna from 2004 to 2021. Patients were divided into two groups: G1 involves all children treated for Pleural Empyema with the traditional approach (thoracic drainage and fibrinolysis), G2 those treated with VATS. We evaluated demographic data, recovery data including clinical data, laboratory tests and imaging exams, surgical findings (surgical technique and operative time, stage of empyema, microbiological analysis, intra-operative complications, conversions), postoperative management (duration of fever, time of stay, days of antibiotic therapy, duration of postoperative drainage, postoperative complications, laboratory and imaging tests performed after surgery, redo surgery) and short and long-term follow-up.

### Surgical techniques

- Pleural drainage and fibrinolysis
  Patients were placed in a semi-sitting position (45°–65°) with a lateral decubitus under general anesthesia. Primary chest tube was placed under US guidance through a 1 cm open incision on the 4–5th intercostal space.
- Video-assisted thoracic surgery (VATS)
  All patients treated with VATS underwent general anesthesia and they were all placed in a lateral decubitus. 3- or 5-mm trocars were used, depending on the patient’s age and weight. First trocar, for the camera, was usually US-guided placed in the 6th intercostal place in the mid axillary line. Low-pressure CO_2_ (2–3 mmHg) was then insufflated to create a working space sufficient to perform the adhesiolysis and the removal of fibrinous material starting from the apex of the lungs and proceeding towards the diaphragm. The working port was placed in the 3rd intercostal space in the posterior axillary line. If surgeons chose to use only one trocar, it was placed using the camera’s trocar (Fr depending on children’s age and weight); instead in case of two trocars: the first one was placed posterior and basal (posterior costo-phrenico sinus), the second one anterior and apical. In all cases, fibrin, pus and all the removed fluids were sent for microbiological analysis. A washing with warm saline solution was done and one or two chest tubes were left in the end of the procedure.

### Statistical analysis

All statistical analyses are performed using *graph-pad* and *r*. Continuous variables are presented as mean, median and standard deviation. Categorical variables are presented as frequency and percentage. A *p* value < 0.05 is considered significant.

## Results

### Demographic data

**Fig.1:**
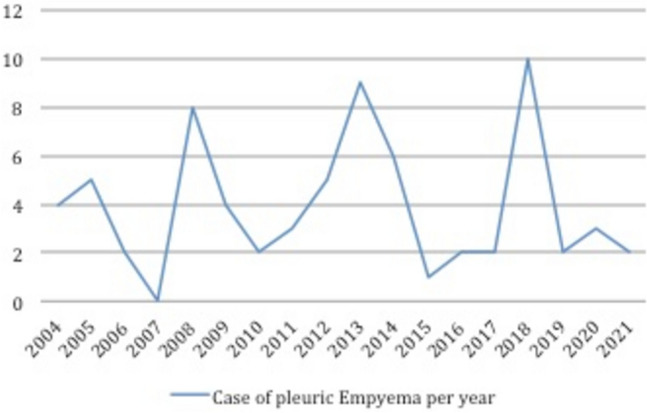
Trend in prevalence of pleuric empyema in pediatric patients involved in the study

70 patients met the inclusion criteria: 40 male (57.2%) and 30 female patients (42.8%) with a mean age of 4.8 years [0, 16]. During the study period, we did not observe a significant trend in the prevalence of empyema (Fig. 1). Vaccination were correctly done for all patients, 40 of them (57.1%) underwent vaccinations for *Pneumococcus spp*, specifically 18 (25.7%) received only PCV7 vaccine, 19 (27.1%) received only PCV13 vaccine and 11 (15.7%) were vaccinated before for PCV7 and then for PCV13. Demographic data of the patients are shown in Table 1. G1 included 12 patients (17.1%) treated with thoracic drainage, G2 58 patients (82.9%) treated with VATS. In G1, 58.3% were male with a mean age at surgery of 4.6 years [0, 12]; while in G2, 56.9% were male, with a mean age of 4.8 years [0, 16].

**Table 1:** Demographic data (age, gender and vaccination done at the time of intervention)

Demographic data	
Age (y); median	4
Gender, *n* (%)	
Male	40 (57.2)
Female	30 (42.8)
Vaccine,* n* (%)	40 (57.1)
Vaccine PCV 7, *n* (%)	18 (25.7)
Vaccine PCV 13, *n* (%)	19 (27.1)
PCV 7 and 13, *n* (%)	11 (15.7)

### Recovery data

The most common symptoms were pyrexia (> 38 °C), cough, dyspnea and thoracic pain. 100% had pyrexia at diagnosis time, 84.3% had cough, 67.1% suffered from dyspnea and 28.6% had thoracic pain. In G1 group, 91.6% had cough, 75% suffered from dyspnea and 25% had thoracic pain, while in G2 group, 83% had cough, 65.5% suffered from dyspnea and 29.3% had thoracic pain (Table 2).

**Table 2:** Recovery data (presenting symptoms)

Presenting symptoms	Tot	G1	G2
Pyrexia (> 38 °C), *n* (%)	70 (100)	12 (100)	58 (100)
Cough, *n* (%)	58 (82.9)	11 (91.6)	48 (83)
Dyspnea, *n* (%)	47 (67.1)	9 (75)	38 (65)
Thoracic pain, *n* (%)	20 (28.6)	3 (25)	17 (29.3)

Routine preoperative laboratory tests showed increased acute inflammatory markers: leukocytosis with a mean value of 17.2 × 10^3^/mm^3^ [4.4, 32.8], neutrophilia with a mean value of 81% [72.8, 83]. CRP was elevated in all cases with a mean value of 13.4 mg/dL [7.3, 23.1]. Specifically in G1, the mean value of leukocytosis was 17 × 10^3^/mm^3^, the mean value of neutrophilia was 78.1% and the mean value of CRP was 12.9 mg/dL; while in G2, the mean value of leukocytosis was 17 × 10^3^/mm^3^, the mean value of neutrophilia was 79.5% and the mean value of CRP was 14 mg/dL (Table 3). 70 patients (100%) underwent Chest X-Ray, 64 patients (91.4%) were studied with Ultrasound too and Chest CT were performed in 13 cases (18.6%) (Table 4). Left-sided empyema was found in 40 children (57.1%), while 30 children (42.9%) had right-sided empyema. No cases of bilateral empyema were observed. In particular in G1, there were 5 cases (41.6%) of left-sided empyema and 7 cases (58.4%) of right-sided empyema; while in G2, there were 35 cases (60.4%) of left-sided empyema and 23 cases (39.6%) of right-sided one.

**Table 3:** Recovery data (laboratory data)

Laboratory data	Tot	G1	G2
Leucocytes, median (10^3^/mm^3^)	16	13.8	15.8
Neutrophils, median (%)	79.3	76	78.3
PCR, median (mg/dL)	12.6	12.5	13.2

**Table 4:** Recovery data (imaging tests)

Imaging tests	Tot
X-Ray, *n* (%)	70 (100)
US, *n* (%)	64 (91.4)
CT, *n* (%)	13 (18.6)

### Surgical findings

Out of the total number of patients included in the study, pleural empyema was intra-operatively classified as stage I in 18 patients (25.7%), as stage II in 30 patients (42.9%) and as stage III in 22 patients (31.4%). In G1, pleural empyema was intra-operatively classified as stage I in 3 patients (25%), stage II in 5 (41.7%) and as stage III in 4 patients (33.3%), whereas in G2, 15 patients (25.9%) were classified as as stage I in, 25 patients (43.1%) as stage II and 18 patients (31%) as stage III (Table 5).

**Table 5:** staging of pleural empyema, in patients treated with Thoracic drainage and fibrinolysis (G1) and with VATS (G2)

Stage of empyema, *n* (%)	Tot	G1	G2
Stage I	18 (25.7)	3 (25)	15 (25.9)
Stage II	30 (42.9)	5 (41.7)	25 (43.1)
Stage III	22 (31.4)	4 (33.3)	18 (31)

During the procedures, thoracic fluids were collected and cultural exam was done, only in 40 (57%) cases, it was possible to perform a microbiological diagnosis, thanks to cultural exams in 21 patients (30%) and PCR in other 37 patients (52,8%); 18 (25.7%) patients received both cultural and PCR diagnosis. Regarding microbiological analysis, in G1, only 1 case (8.3%) of Streptococcus pneumoniae was isolated; in G2, Streptococcus pneumoniae was identified in 14 cases (24.1%), Staphylococcus aureus in just 1 case (1.7%) and Mycoplasma pneumoniae in another 1 case (1.7%), all the other samples were negative. The median surgical time was 45 min in G1 group, 90 min in G2 group. There were no cases of conversion.

### Postoperative management

The mean duration of thoracic drainage was 7.3 days in G1, 6.2 days in G2. In particular, in G1 the mean duration of thoracic drainage was 6 days in stage I, 6.8 days in stage II and 9 days in stage III; while in G2 it was 4.7 days in stage I, 6.48 days in stage II and 7.3 days in stage III.

Patients became afebrile after a mean of 6.4 days in G1 group, 3.9 days in G2 group. Specifically, in G1, the mean duration of fever was 6.6 days in stage I, 5.5 in stage II and 9.5 days in stage III; while in G2, 2 days in stage I, 5.3 days in stage II and 4 days in stage III.

The mean duration of antibiotic therapy was 27.8 days in G1 group and 25 days in G2 group. In particular, in G1, the mean duration of antibiotic therapy was 29 days in stage I, 23.8 days in stage II and 32 days in stage III; while in G2, it was 25.6 days in stage I, 25.4 days in stage II and 24.2 days in stage III.

After surgery, all patients (100%) underwent a controlled Chest X-ray: in G1 group, the median of postoperative X-Ray was 4, instead in G2 group, it was 1.4. Specifically the median of post-operative X-ray was 3.3 in G1 stage I, it was 4.4 in G1 stage II and 5.2 in G1 stage III. In G2 stage I, stage II and stage III the median of post-operative X-ray was 2.

The mean duration of postoperative hospital stay was 16 days in G1 group and 12.1 days in G2 group. In particular, in G1, the mean duration of postoperative hospital stay was 15 days in stage I, 16.6 days in stage II and 16 days in stage III; while in G2 it was 13.5 days in stage I, 12.9 days in stage II and 10 days in stage III.

There were four cases (33.3%) of postoperative complications in G1 group; all subcutaneous emphysema, specifically one case in stage I (33.3%), two in stage II (40%) and one in stage III (25%). As for the G2 group, there were 2 cases (3.4%) of pneumatocele, 1 case (1.7%) of bronchopleural fistula and 14 cases (24.1%) of subcutaneous emphysema. Specifically, there were 1 case (7%) of pneumatocele and 1 case (7%) of bronchopleural fistula in stage I for a total number of 2 cases (14%) of postoperative complications in stage I, 5 cases (20%) of emphysema in stage II and 1 case (5%) of pneumatocele and 9 cases (50%) of emphysema in stage III for a total number of 10 cases (55%) of postoperative complications in stage III.

Among 12 patients included in G1, 2 of them needed a second surgery with VATS with a recurrence rate of 16.7%. Both of them were at II stage pleural empyema.

**Fig. 2:**
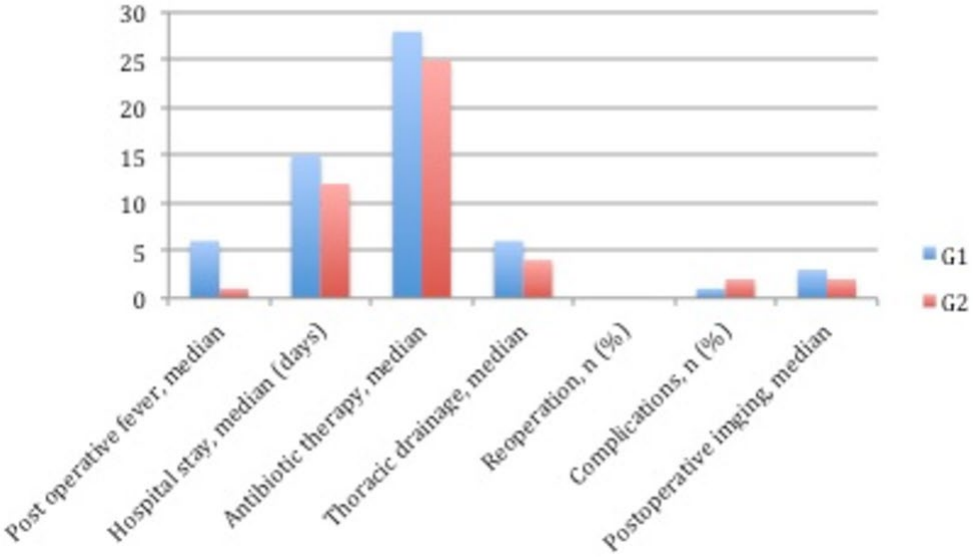
Comparison of outcomes between G1 and G2 groups in stage I

**Fig. 3:**
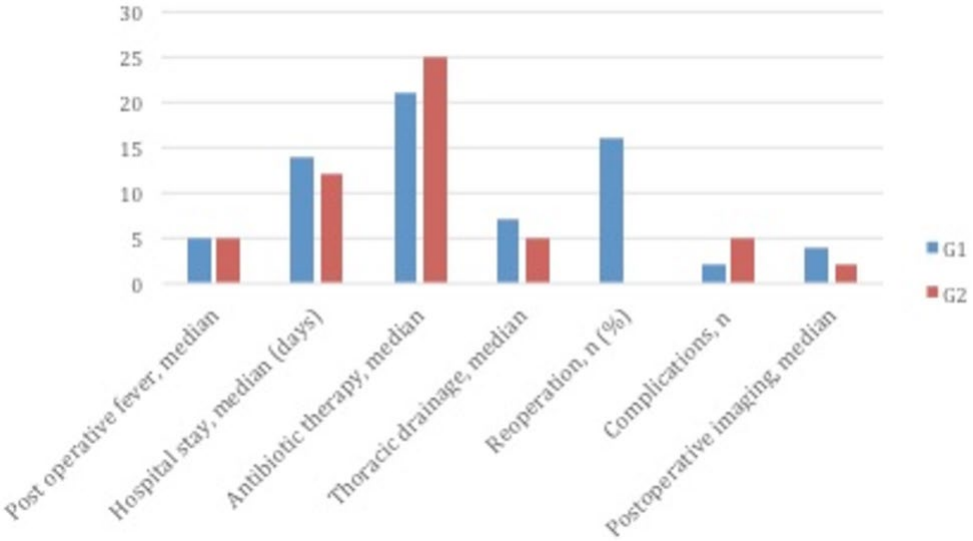
Comparison of outcomes between G1 and G2 groups in stage II

**Fig. 4:**
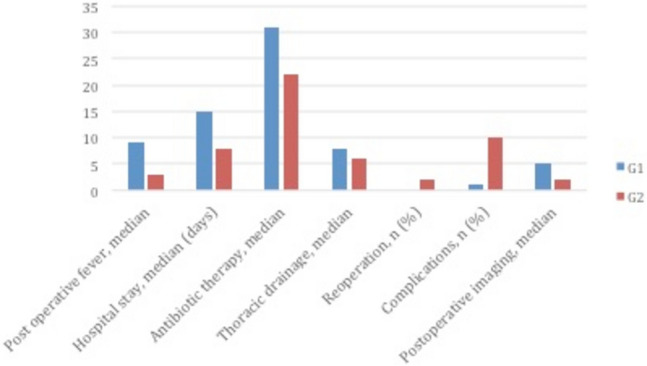
Comparison of outcomes between G1 and G2 groups in stage III

Among 58 patients included in G2, re-operation was performed just in one case (1.7%) because of unsuccessful VATS (stage III); thoracotomy and an atypical lung resection were performed 28 days after. All the post-operative outcomes are presented in Table 6 and they were described according to the stages in Figs. 2, 3 and 4.

**Table 6:** Outcomes of Thoracic drainage (G1) and with VATS (G2)

Outcomes	G1	G2
Duration of postoperative fever, median (days)	5	3
Length of hospital stay, median (days)	15	10.5
Duration of parenteral antibiotics, median (days)	28	25
Duration of thoracic drainage, median (days)	6.5	5
Reoperations, *n* (%)	2 (16.7)	1 (1.7)
Complications, *n* (%)	4 (33.3)	26 (44.8)
Postoperative X-ray, median (days)	4	1.4

### Short and long-term follow-up

Outcomes of treatment of pleural empyema were analyzed in terms of relapse of symptoms and readmission to hospital. Outpatients’ check-ups were performed 1 week, 1 month and 3 months after surgery, respectively, and 100% of patients referred to good general condition.

## Discussion

Pleural Empyema is associated with a high mortality and morbidity rate, despite of different existing treatments [6] such as thoracic drainage with fibrinolysis and minimally invasive treatment consisting of VATS. There is still no consensus on the Gold Standard therapeutic strategy in pediatric age [7].

In our study, the most affected age group by pleural empyema was 3–5 years with a median of 4 years in accordance with the data reported in Literature [9]. Fever was reported to be the most common presenting symptom[8]. All our patients had fever at admission time, cough was present in 84% of patients, dyspnea in 66.7% and thoracic pain in 27.5%. There were no significant differences in terms of presenting symptoms between G1 and G2 patients (*p* > 0.05) demonstrating that clinical features did not interfere with the outcomes of two adopted procedures.

In our analysis, routine laboratory tests evidenced an increased level of inflammations’ markers such as leukocytes (median value of 16 × 10^3^/mm^3^), neutrophils (median value of 79.3%) and CRP (median value of 12.6 mg/dL) with no significant differences between the two groups (*p* > 0.05). All 69 patients were submitted to X-Ray. As shown in Grisaru-Soen et al., the majority of the control group (92%) had a unilateral, unilobar infiltrate on chest X-ray [8]. 100% of our cases were unilateral, specifically in 40 patients (57.1%) pleural empyema was localized in left hemithorax, whereas in 30 patients was in the right one (42.9%) with no statistically significant differences in the whole group of patients (*p* > 0.05). Literature confirmed our results: in Barglik et al., the location of empyema occurred with a similar frequency in both pleural cavities[10]. In 92.7% of patients, chest US was performed too. Literature confirmed the importance of US not only in the diagnosis and the staging of Pleural Empyema, but also, in choosing the correct treatment and the timing for drainage removal. In our centers, the drainage was removed when fluid production was reduced, no air came out and the US did not detect residual pleural sacs.

Stage of pleural empyema was determined intra-operatively depending on the nature of the effusion, the eventual presence of septations and loculations fibrous adhesions of the parietal and pulmonary pleura. In particular, among G1 in 3 patients (25%) pleural empyema was classified as stage I, 5 patients (41.7%) as stage II and 4 (33.3%) as stage III; whereas among all the patients of G2 in 15 patients (25.9%) pleural empyema was classified as stage I, 25 as stage II (43.1%) and 18 patients (31%) as stage III with no statistically significant differences between G1 and G2 (*p* > 0.05). Comparing the operative time, this was significantly reduced in the first group (G1) than in the second (G2): the median surgical time was 45 min in the G1 group and 90 min in the G2 group (*p* < 0.05). Our experience was partially confirmed by Literature data showing a shorter surgical time associated with the traditional approach than that associated with VATS which varied from 80 to 113 min [12, 13]. In Aziz et al. study, surgical time changed depending on the stage For the patients in the I and II phase, the mean was 44 min and for those in the III stage, it was 59 min [14]. Same results were found in our study.

The mean duration of postoperative thoracic drainage in the Literature varied between 3 and 9 days [4, 13, 14]. In our study, the mean duration of thoracic drainage was 7.3 days with a median of 6.5 days in G1, while the mean time of thoracic drainage was 6.2 days with a median of 5 days in G2. This difference in terms of duration of post-operative thoracic drainage was significantly shorter in G2 group than in G1 reducing both the risk of drainage-related infections and time of hospital stay, but not with a statistical significance (*p* > 0.05). As literature showed [15], also in our study, time of drainage was shorter in children operated in earlier stages of empyema.

Duration of postoperative fever in literature ranges from 2 to 5 days [4, 13, 14]. Duration of fever was significantly shorter (*p* < 0.05) in children who underwent VATS than in those treated with thoracic drainage. Moreover, literature showed duration of fever was shorter in children operated in earlier stages of empyema than in those affected by II and III stage pleural empyema as shown in our analysis [15].

Another important advantage associated with the use of VATS was the significant reduction of the length of antibiotic therapy (*p* < 0.05). These data were partially confirmed by Literature showing an important difference in terms of duration of antibiotic therapy between VATS and drainage but with different values. In Scarci et al. [16], the average length of antibiotic therapy was 12,8 days for VATS, 21,3 days for the traditional approach. Cohen et al. showed very similar results: 7.6 days for VATS, 18.2 for chest drainage [17]. Comparing these results with the mean duration of antibiotic therapy for VATS and drainage in our study, a higher result is evident. This is probably due to our decision to continue antibiotic therapy also at home. According to Literature review [10], also in our analysis, there was no statistically significant difference in term of duration of antibiotic therapy between the three different stages, respectively, in G1 and G2.

Number of postoperative X-ray was higher in G1 than in G2 (*p* < 0.05), so in patients treated with VATS, exposition to radiation was lower than in those treated with a traditional approach. VATS allowed us also a shorter time of hospitalization than thoracic drainage (*p* < 0.05) probably because of the reduced time of fever, the reduced duration of drainage, the reduced number of post-operative X-ray and the the lower rate of reoperation. Median duration of hospital stay in pediatric patients affected by pleural empyema and treated with primary VATS in literature was shorter than that of patients treated with drainage, as we showed, but it ranged from 4.6 to 16.4 days (median 7 days) [4, 13, 14]. Specifically, in 2011, Scarci et al. an average length of stay of 10,8 days was associated to VATS and 20 days to the traditional approach [16]; in Kurt et al. [18], in Aziz et al. [14] and in Li and Gates [19], we could find similar results. Literature also showed a significantly shorter hospital stay in children operated in earlier stages [4, 14]. Our data showed the same differences. About complications, there were no cases of intraoperative complications neither in G1 nor in G2 groups, but there were different cases of postoperative complications. Children treated in stages I and II showed significantly better postoperative results compared with the children treated in stage III [15]. Redo-surgery rate is significantly higher in G1 than in G2 (*p* < 0.05) with no differences among the three stages. Literature completely confirmed our results: Avansino et al. study reported a re-intervention ratio in VATS of 2.5% and a second surgery ratio in thoracic drainage of 23.5% [20]. A considerable portion of the patients who underwent primary chest tube treatment later required VATS-assisted pleural debridement, leading to a longer ICU stay and significantly prolonged hospitalization [14].

## Conclusion

Even if it is a retrospective study, it clearly provides the superiority of VATS-assisted pleural debridement rather than thoracic drainage as primary therapy for pleural empyema in pediatric patients. This is not affected by demographical data, presenting symptoms or initial laboratory tests which are essentially the same between G1 and G2 groups.

VATS-directed therapy is an effective and safe procedure which allows precise placement of the chest tube in addition to thorough removal of pleural fluid and peel on the surface of the lung. Moreover, it is associated with some important advantages such as the reduction of chest tube drainage, duration of fever, hospital stay, time of antibiotic therapy and recurrence rate.

### Supplementary Information

Below is the link to the electronic supplementary material.

Supplementary file1 (DOCX 131 KB)

Supplementary file2 (DOCX 21 KB)

## Data Availability

The data that support the findings of this study are available on request from the correspondig author.

## Abbreviations

VATS: Video assisted thoracic surgery
US: Ultrasound
CT: Computed tomography
G1: Group 1
G2: Group 2
