# Supplementary material for: Pleural drainage vs video-assisted thoracoscopic debridement in children affected by pleural empyema
Source: Pediatr Surg Int. 2023 Nov 6;39(1):287. doi: 10.1007/s00383-023-05566-z (PMC10625948; doi:10.1007/s00383-023-05566-z)
Supplement: Supplementary file 1 — Supplementary file1 (DOCX 131 KB) [file 383_2023_5566_MOESM1_ESM.docx]

**List of Figures**

Figure 1

*Fig.1 Trend in prevalence of pleuric empyema in pediatric patients involved in the study from 2004 to 2021.*

Figure 2

*Fig. 2: Comparison of outcomes between G1 and G2 groups in stage I of pleural empyema*

Figure 3

*Fig. 3: Comparison of outcomes between G1 and G2 groups in stage II of pleural empyema*

Figure 4

*Fig. 4: Comparison of outcomes between G1 and G2 groups in stage III of pleural empyema*
