# Supplementary material for: Pleural drainage vs video-assisted thoracoscopic debridement in children affected by pleural empyema
Source: Pediatr Surg Int. 2023 Nov 6;39(1):287. doi: 10.1007/s00383-023-05566-z (PMC10625948; doi:10.1007/s00383-023-05566-z)
Supplement: Supplementary file 2 — Supplementary file2 (DOCX 21 KB) [file 383_2023_5566_MOESM2_ESM.docx]

**List of Tables**

Table 1

| *Demographic data* | | |
| --- | --- | --- |
| *Age (y); median* | | *4* |
| *Gender,n (%)* | *Male* | *40 (57.2)* |
|  | *Female* | *30 (42.8)* |
| *Vaccine, n (%)* | | *40 (57.1)* |
| *Vaccine PCV 7 , n (%)* | | 18 (25.7) |
| *Vaccine PCV 13, n (%)* | | 19 (27.1) |
| PCV 7 and 13, n (%) | | 11 (15,7) |

*Table 1: Demographic data (age, gender and vaccination done at the time of intervention)*

Table 2

| *Presenting symptoms* | *Tot* | | *G1* | *G2* |
| --- | --- | --- | --- | --- |
| *Pyrexia (>38°C), n (%)* | | *70 (100)* | *12 (100)* | *58 (100)* |
| *Cough, n (%)* | | *58 (82.9)* | *11 (91.6)* | *48 (83)* |
| *Dyspnea, n (%)* | | *47 (67.1)* | *9 (75)* | *38 (65)* |
| *Thoracic pain, n (%)* | | *20 (28.6)* | *3 (25)* | *17 (29.3)* |

*Table 2: Recovery data (presenting symptoms)*

Table 3

| *Laboratory data* | Tot | | | G1 | G2 |
| --- | --- | --- | --- | --- | --- |
| *Leucocytes, median (10^3^/mm^3^)* | | *16* | *13.8* | | *15.8* |
| *Neutrophils , median (%)* | | *79,3* | *76* | | *78.3* |
| *PCR, median (mg/dL)* | | *12.6* | *12.5* | | *13.2* |

*Table 3: Recovery data (laboratory data)*

Table 4

| *Imaging tests* | Tot |
| --- | --- |
| *X-Ray, n (%)* | *70 (100)* |
| *US , n (%)* | *64 (91.4)* |
| *CT, n (%)* | *13 (18.6)* |

*Table 4: Recovery data (imaging tests)*

Table 5

| *Stage of empyema, n (%)* | *Tot* | *G1* | *G2* |
| --- | --- | --- | --- |
| *Stage I* | *18 (25.7)* | *3 (25)* | *15 (25.9)* |
| *Stage II* | *30 (42.9)* | *5 (41.7)* | *25 (43.1)* |
| *Stage III* | *22 (31.4)* | *4 (33.3)* | *18 (31)* |

*Table 5: staging of pleural in all patients included in the study, in patients treated with Thoracic drainage and fibrinolysis (G1) and in patients treated with VATS (G2)*

Table 6

| *Outcomes G1* | | *G2* |
| --- | --- | --- |
| *Duration of postoperative fever, median (days)* | *5* | *3* |
| *Length of hospital stay, median (days)* | *15* | *10.5* |
| *Duration of parenteral antibiotics, median (days)* | *28* | *25* |
| *Duration of thoracic drainage, median (days)* | *6.5* | *5* |
| *Reoperations, n (%)* | *2 (16.7)* | *1 (1.7)* |
| *Complications, n (%)* | *4 (33.3)* | *26 (44.8)* |
| *Postoperative X-ray, median (days)* | *4* | *1.4* |

*Table 6: outcomes of treatment in patients treated with Thoracic drainage and fibrynolisis (G1) and in patients treated with VATS.*
